# Preferences for Attention-Deficit/Hyperactivity Disorder (ADHD) Non-Stimulant Treatment Characteristics Among Children and Adolescents With ADHD and Their Caregivers

**DOI:** 10.36469/9842

**Published:** 2016-10-28

**Authors:** Emuella Flood, Kavita Gajria, Vanja Sikirica, Paul Hodgkins, M. Haim Erder, Frank Lopez, Daniel Connor

**Affiliations:** 1 Commercialisation and Outcomes, ICON PRO, Bethesda, MD, USA; 2 Global Health Economics, Outcomes Research and Epidemiology, Shire, Wayne, PA, USA; 3 Private Practice, Children’s Development Center P.A., Winter Park, FL, USA; 4 Division of Child & Adolescent Psychiatry University of Connecticut Health Center, Farmington, CT, USA

**Keywords:** caregivers, children/adolescents, medication preferences, adaptive conjoint analysis, attention-deficit/hyperactivity disorder

## Abstract

**Background:** Understanding patient and caregiver preferences for treatment is important for optimizing treatment decisions. Non-stimulant therapies are an alternative treatment option to stimulant therapy for attention-deficit/hyperactivity disorder (ADHD). Guanfacine extended release (GXR) and atomoxetine (ATX) are two non-stimulant medications approved in the United States for the treatment of ADHD.

**Objective:** To identify non-stimulant ADHD medication attributes important to caregivers/patients.

**Methods:** US caregivers of ADHD patients (6–17 years) and child/adolescent patients (10–17 years) completed an adaptive conjoint analysis survey. Respondents selected between hypothetical treatments with different attributes. Ordinary least-squares and hierarchical Bayes regression using Sawtooth Software were used to calculate utilities, importance ratings, and preferences.

**Results:** 483 caregivers (mean age: 41.9 years, standard deviation [SD]: 8.7; 75% female) and 211 children/adolescents (mean age: 14.5 years, SD: 2.2; 70% male) completed the survey. Based on importance ratings, the most influential attributes for both caregivers and children/adolescents were chance of somnolence, efficacy, and for caregivers, effect on oppositionality and black box warning. Most caregivers (95.3%) and children/adolescents (93.8%) preferred GXR over ATX. In several sensitivity analyses in which attribute levels varied, GXR remained the preferred medication with the exception of one scenario.

**Conclusions:** Children/adolescents and caregivers demonstrated in this study that they can clearly express their preferences for treatment attributes and treatment choices; in this case they preferred GXR to ATX. Patients and caregiver preferences could be useful inputs to the treatment selection decision-making process.

## BACKGROUND

The current literature on patient care has shown a gradual shift from a paternalistic model, which assumes that the patient serves a passive role, to a model of shared decision making, in which patients and caregivers are active participants in their healthcare plans.[1-4] A number of studies have suggested that this approach, which emphasizes high patient or caregiver involvement in treatment decisions, results in decreased healthcare expenditures and increased satisfaction, adherence, and health-related quality of life (HRQoL) outcomes.[5-8] The US government has also recognized the need for a more patient-centered approach to medicine: the Patient-Centered Outcomes Research Institute (PCORI), which emphasizes the role of the patient in informed decision making, was founded as part of the Patient Protection and Affordable Care Act in 2010.[9] As practitioners are increasingly advocating for this patient-focused model and encouraging patients and caregivers to become more active partners in their treatment plans, it has also become increasingly important to understand patient and caregiver preferences with respect to aspects of treatment, and to ensure that patients and caregivers are provided with adequate information about these aspects so they can make informed decisions.

Although the shared decision-making model has been studied extensively within the healthcare field, only recently has it been examined specifically within the context of mental health.[10] However, evidence suggests that this model shows promise across a broad range of psychiatric conditions, among both patients and their caregivers.[11,12] Attention-deficit/hyperactivity disorder (ADHD) is one of the most common childhood disorders, affecting an estimated 7% of children in the United States.[13] Symptoms of ADHD can include hyperactivity, impulsivity, and difficulties with sustained attention, and the disorder is associated with a substantial decline in the child's HRQoL, particularly with regard to psychosocial functioning.[14] It is also often associated with comorbid conditions, such as learning disabilities, oppositional defiant disorder, conduct disorder, mood disorders, anxiety disorders, obsessive-compulsive disorder, Tourette's syndrome, substance abuse disorders, and sleep disorders.[15–18]

The most common medications used to treat ADHD are stimulants, such as methylphenidate and amphetamine and their derivatives.[19] Several studies have successfully elicited patient and caregiver preferences for these medications.[20,21] However, while these medications are generally highly effective at reducing symptoms, some patients do not experience adequate symptom control, stimulants also have been associated with sleep problems, decreased appetite, anxiety, irritability, and headache.[22] Furthermore, they are controlled substances, and for some caregivers there is reluctance to use these treatments for their children because of perceived safety concerns, even though they are generally safe and well tolerated. In addition, it has been demonstrated that such medications have the potential for abuse in some patients.[23]

Non-stimulant therapies are an alternative treatment option to stimulant therapy.[24,25] Guanfacine extended release (GXR) and atomoxetine (ATX) are two non-stimulant medications that have regulatory approval for use in ADHD within the United States. ATX is a selective norephinephrine reuptake inhibitor (SNRI) that, unlike stimulants, does not directly target dopamine.[26] GXR is a 'first in class' $\alpha_{2A}$-adrenoceptor agonist that aims to improve attention by targeting receptors in the prefrontal cortex and the locus coeruleus involved in the synthesis of norepinephrine. Both medications vary with respect to efficacy, regimen, and risk of side effects.[27-29]

Patient preference refers to a patient's perception of the value for alternative treatment options when presented with information about their risks and benefits.[30] A few studies have assessed caregiver and patient preferences for ADHD treatment-related health states or attributes associated with stimulant medications specifically[31,32]; however, no studies have examined drivers of treatment preferences for non-stimulant medications specifically among caregivers or patients, and no studies have examined preferences from the perspective of children with ADHD. The objective of this study was to identify relevant treatment attributes that drive treatment preferences for non-stimulants among caregivers and children/adolescents with ADHD. A second objective was to assess preference for GXR versus ATX in caregivers and children/adolescents with ADHD based on their specific treatment attributes.

## METHODS

This was a cross-sectional study based on a one-time, Internet-based survey completed by both caregivers with a child/adolescent (aged 6–17 years) with ADHD and by children/adolescents (aged 10–17 years) with ADHD. Study participants were recruited through a market research panel with expertise in recruiting for survey studies. The panel was constructed using a process called "address-based sampling" (randomly selecting residential addresses to invite to join), and was thus representative of a diverse sample of more than 50,000 US households. Members were asked to identify any health conditions within their households so that they could be targeted for relevant research studies on those reported health conditions.

For this study, caregivers with a child/adolescent aged 6–17 years and a caregiver-reported diagnosis of ADHD completed the online survey. The children/adolescents of some of these caregiver respondents also participated in the study. Thus, some caregiver respondents were part of a caregiver/child dyad in which the caregiver and their child participated, whereas other caregivers were not part of a dyad and did not have their child participate. All child/adolescent respondents had a caregiver who participated, and thus all child/adolescent respondents were part of a dyad. These children/adolescents were aged 10–17 years and had a caregiver-reported physician diagnosis of ADHD. Children/adolescents were only allowed to participate with their parents' consent. All participants were screened online to ensure that they met the specific eligibility criteria (Figure 1). In particular, caregivers were required to have written and oral fluency in English, at least a fourth grade reading level, and access to internet connectivity to complete the web-based survey. Caregivers with self-reported dementia, Alzheimer's disease, schizophrenia, or schizophrenic disorder were not eligible to participate. In addition, children and adolescents with a caregiver-reported diagnosis of schizophrenia, schizophrenic disorder, or mental retardation (<70) were not eligible to participate. Actual experience with the two non-stimulants being evaluated, GXR and ATX, was not an eligibility requirement.

Participants completed the survey at home. Both the caregivers and the children/adolescents who completed the survey were compensated for their time in the form of participation points, which could be redeemed for various goods and services, equaling $30 per respondent. The study followed the Declaration of Helsinki, and the protocol was approved by an institutional review board (Schulman IRB; Plantation, FL, USA). This research was implemented following published methodological guidelines for conjoint studies.[26] The survey design and analysis are detailed below.

### Conjoint Analysis

Caregiver and child/adolescent preferences were elicited by an adaptive conjoint analysis (ACA) survey. Conjoint analysis, which is increasingly being used in evaluating medical interventions, involves respondents making trade-offs among treatment features (called attributes) and has been used in a number of studies to assess patient preferences for treatment of chronic conditions.[33,34] The resulting data enable the assessment of the relative importance of each attribute; specifically, they show the influence that each attribute has on overall treatment preferences. Based on the relative importance of each attribute, we were also able to evaluate the estimated percentages of caregivers and children/adolescents who would prefer each of the two non-stimulants, GXR and ATX; how a change in a product attribute, such as better efficacy or less risk of side effects, impacts the estimated preference for a treatment; how preferences for non-stimulants compare between caregiver and children/adolescents among caregiver/child dyads; and how preferences for non-stimulant treatment options may differ between selected subgroups of caregivers and/or children/adolescents.

**Figure 1.** Inclusion and Exclusion Criteria for Caregivers and Children/Adolescents

| Inclusion criteria | Exclusion criteria |
|---|---|
| *Caregiver*<br>• Parent/caregiver with a child/adolescent between the ages of 6 and 17 years with a physician diagnosis of ADHD<br>• Written and oral fluency in English<br>• Grade 4 or higher reading level in English<br>• Willing and able to provide written informed consent<br>• Access to an Internet connection to complete the web-based survey | *Caregiver*<br>• Self-reported current diagnosis of dementia, Alzheimer's disease, schizophrenia, or schizoaffective disorder<br>• Legal incapacity that would preclude the participant's participation in or ability to complete the study |
| *Child/adolescent*<br>• Child/adolescent between the ages of 10 and 17 years with a physician diagnosis of ADHD as reported by the caregiver<br>• Written and oral fluency in English<br>• Grade 4 or higher reading level in English<br>• Willing and able to provide written informed consent | *Child/adolescent*<br>• Caregiver-reported current diagnosis of schizophrenia or schizoaffective disorder or mental retardation (<70 IQ)<br>• Legal incapacity that would preclude the participant's participation in or ability to complete the study |

ADHD: attention-deficit/hyperactivity disorder; IQ: intelligence quotient

ACA is one type of conjoint analysis. With ACA, the respondents complete an electronic questionnaire, and the questionnaire is tailored to the respondents based on their answers to previous questions.[33,34] Thus, the survey is adapted to focus on those attributes that the respondent considers most important and those attribute levels regarded as most relevant. In the ACA survey, data were collected in different phases. In the first phase, respondents were asked to rate the levels of each attribute in terms of acceptability on a 7-point scale, from 1, "not at all acceptable," to 7, "acceptable" (caregiver survey), or a 5-point scale, from 1, "I would not like this at all," to 5, "I would like this a lot" (child/adolescent survey). The second phase (paired comparison questions) elicited treatment preferences by asking respondents to make trade-offs among attributes and choose between hypothetical treatments – labeled as Treatment A and Treatment B. These two treatment profiles were presented with different levels of the same attributes. Respondents used a 7-point scale to indicate not only their preference, but the strength of their preference; response options ranged from "strongly prefer A" to "strongly prefer B". The profiles presented to respondents in this second phase were customized based on responses to previous questions. Specifically, the choice tasks became more difficult by focusing on attributes that were most important to the respondent based on responses to previous questions. The final section of the survey contained questions about demographic and clinical history. The survey was pilot-tested with respect to wording and comprehension in two rounds of cognitive interviews with a total of 12 caregivers and 10 children/adolescents with ADHD, and revisions were made to improve clarity.

## Survey Attributes

Thirteen attributes of the two most common non-stimulant treatments for ADHD were identified from a review of the literature on ADHD, clinician input, and the US Food and Drug Administration (FDA)-approved labels for GXR and ATX. These attributes included those related to efficacy, regimen, black box warning, common side effects, and oppositional symptoms. As neither non-stimulant medication is indicated for the treatment of oppositionality, this attribute was included as an attribute to only explore its relative importance in influencing preferences, but was not considered when comparing the overall product preference.

Attributes were described in lay terminology so that they could be easily understood by participants. Clarity and comprehension were assessed during pilot testing with participants matching the target study sample. For each of the two non-stimulants, a product attribute was defined by different levels, such that they represented the full range of possibilities for an attribute (ie, the highest possible level, the lowest possible level, and a middle level). All attributes had three levels, with the exception of a black box warning, which had two: presence or absence of a black box. Thirteen attributes were included in the caregiver survey, of which ten were included in the child/adolescent survey because three attributes (black box warning, blood pressure, and oppositionality) were concepts deemed too difficult to be understood by children/adolescents from pilot testing. Based on the peer-reviewed literature and FDA-approved labels for the target products, attributes, as well as their base-case values, were identified to match the profiles of the two target stimulants. The selection of attributes and base-case values were then reviewed and confirmed by clinical ADHD experts. As neither ATX nor GXR is indicated for treatment of oppositionality, the base-case levels for this attribute were informed only by the literature and clinician input rather than product labels. Table 1 presents the base-case values for all attributes of ATX and GXR.

**Table 1.** Summary of Base-case Values

| Attribute | ATX | GXR |
|---|---|---|
| Black box warning | • Black box warning for suicidal thoughts | • No black box warning for suicidal thoughts |
| Improvement in ADHD symptoms (clinician assessment) | • Your child's doctor says the medication is working moderately well<br><br>• [Children] Your doctor says it is working OK | • Your child's doctor says the medication is working well<br><br>• [Children] Your doctor says it is working well |
| Improvement in ADHD symptoms (teacher assessment) | • Your child's teacher notices the medication is working moderately well<br><br>• [Children] Your teacher says it is working OK | • Your child's teacher notices the medication is working well<br><br>• [Children] Your teacher says it is working well |
| Regimen | • Take one dose daily | • Take one dose daily |
| Onset of effect | • Takes 4–8 weeks to start working | • Takes up to 2 weeks to start working |
| Fatigue | 8% | 14% |
| Nausea | 10% | 6% |
| Abdominal pain | 18% | 10% |
| Decreased appetite | 16% | 5% |
| Somnolence | 11% | 45% |
| Headache | 19% | 24% |
| Change in blood pressure | 5% increase | 6% decrease |

ADHD: attention-deficit/hyperactivity disorder; ATX: atomoxetine; GXR: guanfacine extended release

### Data Analysis

The conjoint data were analyzed utilizing Sawtooth Software (Sequim, WA, USA) v7.0.26 and the logit/hierarchical Bayes module. Patients who had two or more illogical responses (eg, reported that a greater risk of a side effect was more acceptable than a lower risk, or responded incorrectly to a "clear winner" question, ie, a pairs question specifically designed with one profile that all respondents would prefer over the other profile) were excluded from the conjoint analysis.

Analysis of ACA data involved the combination of the initial rating (the acceptability questions in the first phase) and the paired comparison questions (second phase). Specifically, using the information from both the acceptability rating questions and the paired comparisons, utility values (ie, preference weights) were calculated. These preference weights enabled the calculation of the relative importance of each attribute for each respondent in influencing treatment preferences; the percentages of relative importance for each attribute were then individually summed for the total sample to determine which attributes were the primary drivers in hypothetical treatment decisions based on having the highest percentage weight.

### Sensitivity Analyses

Univariate sensitivity analyses were conducted where selected attribute levels were varied and the subsequent impact on treatment preferences was evaluated. Specifically, in addition to the base-case scenario, in which overall preferences for GXR versus ATX were compared based on base-case characteristics, 14 scenarios among the caregivers and 12 scenarios among the children/adolescents were tested. In these scenarios, levels of selected attributes were varied from the base-case and preferences were compared. All of the above conjoint analyses were also stratified by sociodemographic and clinical subgroups, and the student t-test was used to make comparisons between groups. Specifically, we stratified by age (caregivers were stratified by those having a child aged 6–12 years versus those having a child aged 13–17 years; child/adolescent participants were stratified by those aged 10–12 years versus those aged 13–17 years), sex, race (white versus non-white), income level (under $50,000/year versus $50,000 or more/year), education level (college degree versus no college degree), time since diagnosis (more than 2 years versus 2 years or under), comorbidity burden (at least one comorbidity versus no comorbidities), current and previous ADHD treatment (stimulant versus non-stimulant; treatment experienced versus treatment naïve), self-reported severity (mild to moderate versus severe to very severe), and education assistance (extra help with reading versus no extra help with reading). Finally, among caregiver/child dyads, a comparison between caregivers and their children regarding their preferences for GXR versus ATX was conducted. Specifically, a t-test was performed to determine the level of agreement in preferences between the participating caregivers and their children/adolescents.

## RESULTS

A total of 5,422 caregivers completed the online screener, of whom 544 were eligible and 528 proceeded to complete the survey. Of these 528 caregivers, 223 had an eligible child or adolescent, and 221 of these children/adolescents completed the survey. After excluding participants with more than two illogical responses, the effective sample for analysis included 483 caregivers and 211 children/adolescents.

The mean (standard deviation [SD]) age of the caregiver participants was 41.9 (8.7) years and 75% were female. The majority were white (77%), 10% were Hispanic, and 7% were black. Of the caregivers, 60% had less than a bachelor's degree and 58% were employed.

The sociodemographic and clinical characteristics of both the children/adolescents of the caregiver participants and the child/adolescent participants, as reported by the caregivers, are provided in Table 2. For the 483 children/adolescents of caregiver participants, the mean (SD) age was 12.1 (8.9) years and 70% were male. For the 211 child/adolescent participants, the mean (SD) age was 14.5 (2.2) years and 70% were male. As reported by the caregivers, 73% of children/adolescents were currently on medication, with 9% of those currently on GXR or ATX. At the time of survey completion, the mean (SD) time since the child/adolescent had been diagnosed with ADHD was 6.3 (3.6) years. The majority of patients (72%) had moderate to very severe disease as reported by the caregivers. No significant differences in sociodemographic and clinical characteristics were observed between age groups (caregivers of children/adolescents aged 6–12 years versus 13–17 years; and children/adolescents aged 10–12 years versus 13–17 years; $P>0.05$).

Acceptability ratings for each attribute and part-worth utilities were ordered in the direction that was expected, where the most favorable attribute level had the highest rating/utility and the least favorable level had the lowest; this was the case for both caregiver and child/adolescent participants. Again, no differences were observed between age subgroups (10–12 years versus 13–17 years) in either the acceptability ratings or the utilities. The part-worth utilities enabled the computation of the relative importance of each attribute as compared to the other importance levels of all attributes; these values are reported in Figures 2 and 3 for the caregiver and child/adolescent samples, respectively. The attributes that were primary drivers of preference to the caregivers as assessed by the mean relative importance ratings were somnolence (11.0%) and improvement in oppositionality (10.7%); least important were dosing regimen (5.5%) and impact on blood pressure (3.9%). Among the children/adolescents, somnolence (12.3%) and improvement in ADHD symptoms (teacher's assessment: 12.1%; clinician's assessment: 11.5%) were the most important attributes; dosing regimen (8.4%) and chance of headache (7.5%) were least important. The relative importance of attributes is interpreted as linear. They are ratio data, meaning that an attribute with an 8% importance rating is twice as important as an attribute with a 4% importance rating. Thus, for example, among the caregivers, onset of effect (8.1%) was about twice as important as impact on blood pressure (3.9%); among children/adolescents, chance of somnolence (12.3%) was more than 1.5 times as important as chance of headache (7.5%). Levels of relative importance of one attribute versus another were similar between the age subgroups for both caregivers (child aged 6–12 years versus 13–17 years) and children/adolescents (aged 10–12 years versus 13–17 years).

Table 3 presents the estimated percentages of caregivers and children/adolescents preferring each non-stimulant (GXR versus ATX) given the utilities for the base-case attribute levels and also for various sensitivity analysis scenarios. Given the base-case, the majority of both caregivers (95.3%) and children/adolescents (93.8%) showed a clear preference for GXR over ATX; no significant differences were observed between selected sociodemographic and clinical subgroups (age, race, education level, and various aspects of clinical experience), with the exception of race (white versus non-white, $P<0.01$), as a higher percentage of white respondents preferred GXR compared with non-white respondents.

Univariate sensitivity analyses demonstrated that GXR remained the most preferred medication in all scenarios except one in which efficacy was set to equal for both the GXR and ATX profiles, and the levels of each attribute were changed to reflect the worst case for GXR and best case for ATX (based on the range of estimates reflected by the base case). Specifically, there was a strong shift in preference, with 73.7% of caregivers and 60.3% of children/adolescents preferring ATX over GXR. Varying the levels of the black box warning attribute also resulted in a shift in caregiver preference from GXR to ATX, particularly in a scenario in which GXR had a black box warning and ATX did not. Even though GXR was preferred over ATX in all other sensitivity scenarios, the estimated percent of respondents preferring this treatment often decreased substantially (eg, from 93.8% to 61.8%) for the children/adolescents in a scenario in which efficacy as perceived by both teacher and clinician was set to equal for both non-stimulants.

**Table 2.** Demographic and Clinical Characteristics of Child/Adolescent as Reported by Caregiver

| | Children/Adolescents of Caregiver Participants (n=483) | Child/Adolescent Participants (n=211) |
|---|---|---|
| **Sample Characteristics** | | |
| Age, years | | |
| N | 483 | 211 |
| Mean (SD) | 12.1 (8.9) | 14.5 (2.2) |
| Median | 12 | 14 |
| Minimum, maximum | 6, 17 | 10, 17 |
| Sex | | |
| Male | 336 (70) | 148 (70) |
| Female | 147 (30) | 63 (30) |
| Hispanic or Latino | | |
| Total | 481 | 210 |
| Yes | 71 (15) | 28 (13) |
| No | 410 (85) | 182 (87) |
| Racial background | | |
| Total | 483 | 211 |
| White/Caucasian | 423 (88) | 188 (89) |
| Black/African-American | 56 (12) | 21 (10) |
| Asian | 11 (2) | 6 (3) |
| American Indian/Alaska Native | 13 (3) | 8 (4) |
| Native Hawaiian/Pacific Islander | 1 (0.2) | 1 (0.5) |
| **Clinical Characteristics** | | |
| History of ADHD, years | | |
| N | 483 | 211 |
| Mean (SD) | 6.3 (3.6) | 8.0 (3.7) |
| Median | 5 | 8 |
| Minimum, maximum | <1, 9 | 12, <1 |
| Currently taking medication for ADHD at baseline | | |
| Total | 481 | 211 |
| No | 131 (27) | 57 (27) |
| Yes | 350 (73) | 154 (73) |
| Current medication used for ADHD | | |
| Total | 483 | 211 |
| ATX | 14 (3) | 4 (2) |
| GXR | 30 (6) | 9 (4) |
| Stimulant | 316 (65) | 143 (68) |
| None | 135 (28) | 59 (28) |

Notes: Data are numbers (%) unless stated otherwise.

ADHD: attention-deficit/hyperactivity disorder; ATX: atomoxetine; GXR: guanfacine extended release; SD: standard deviation

**Table 2.** Demographic and Clinical Characteristics of Child/Adolescent as Reported by Caregiver - cont'd

| | Children/Adolescents of Caregiver Participants (n=483) | Child/Adolescent Participants (n=211) |
|---|---|---|
| **Clinical Characteristics - cont'd** | | |
| Previous medication used for ADHD | | |
| Total | 483 | 211 |
| ATX | 87 (18) | 50 (24) |
| GXR | 30 (6) | 5 (2) |
| Stimulant | 367 (76) | 173 (82) |
| None | 117 (24) | 34 (16) |
| Severity of child's ADHD | | |
| Total | 482 | 211 |
| Very mild | 23 (5) | 16 (8) |
| Mild | 110 (23) | 52 (25) |
| Moderate | 226 (47) | 100 (47) |
| Severe | 113 (23) | 40 (19) |
| Very severe | 10 (2) | 3 (1) |
| Comorbid conditions of child | | |
| Anxiety | 70 (14) | 28 (13) |
| Autism | 31 (6) | 11 (5) |
| Bipolar disorder | 24 (5) | 16 (8) |
| Conduct disorder | 8 (2) | 5 (2) |
| Depression | 53 (11) | 30 (14) |
| Drug and alcohol abuse | 3 (1) | 0 |
| Learning disability | 81 (17) | 39 (18) |
| Oppositional defiant disorder | 53 (11) | 23 (11) |
| Obsessive-compulsive disorder | 24 (5) | 12 (6) |
| Pervasive developmental disorder | 10 (2) | 0 |
| Schizophrenia | 1 (0.2) | 1 (0.5) |
| Tics disorder | 8 (2) | 6 (3) |
| Tourette's syndrome | 10 (2) | 4 (2) |
| Other | 25 (5) | 10 (5) |
| None of the above | 256 (53) | 112 (53) |
| Comorbidity burden | | |
| At least one comorbidity | 227 (47) | 99 (47) |
| No comorbidities | 256 (53) | 112 (53) |
| Number of comorbidities | | |
| Mean (SD) | 0.8 (1.3) | 0.8 (1.3) |
| Median | 0 | 0 |

Notes: Data are numbers (%) unless stated otherwise.

ADHD: attention-deficit/hyperactivity disorder; ATX: atomoxetine; GXR: guanfacine extended release; SD: standard deviation

**Figure 2.** Mean Percent Importance Ratings for Attributes among Caregivers

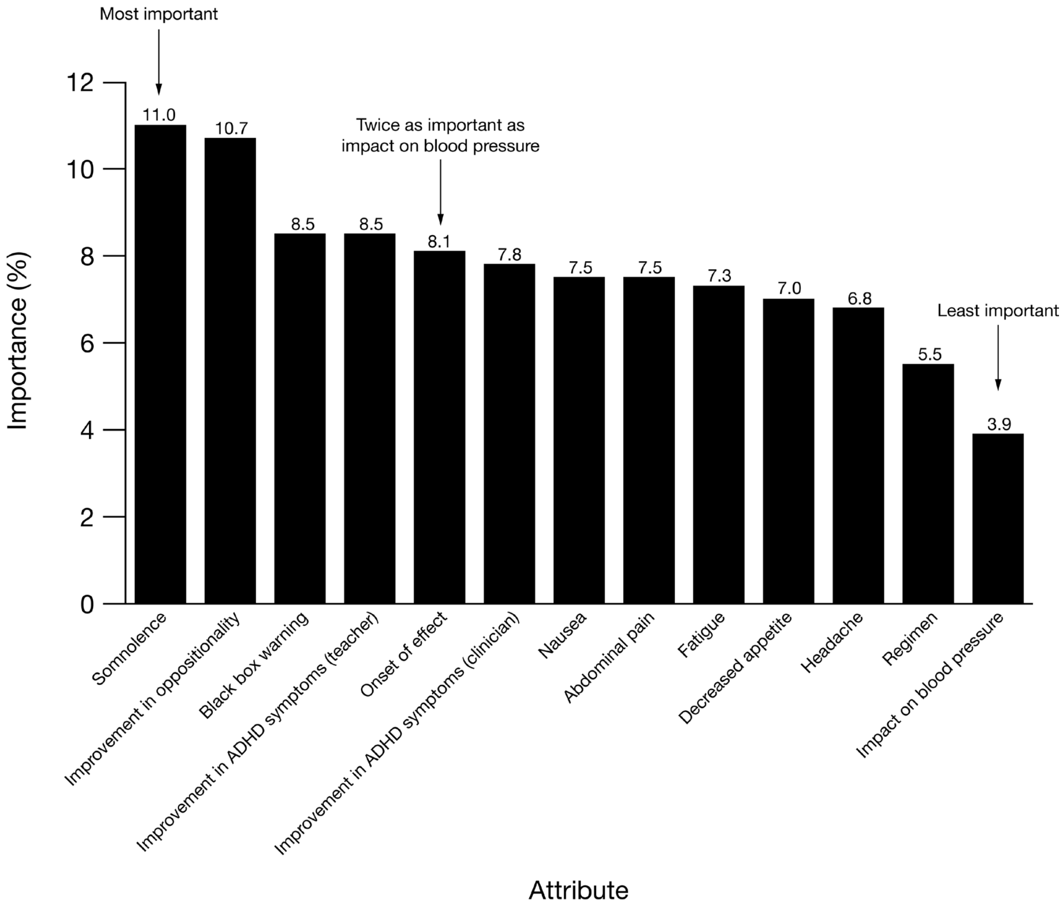

ADHD: attention-deficit/hyperactivity disorder

In the dyads analysis, a total of 97.3% of caregiver-child/adolescent dyads were in agreement with respect to their preference for a treatment profile, with the majority agreeing that they would prefer GXR. Table 4 compares the rank order of the relative importance of individual attributes levels between the caregivers and children in dyads (considering only the attributes common to both samples). Somnolence and improvement in ADHD symptoms (teacher assessment) were ranked 1 and 2, respectively, in terms of importance in both samples. Third most important was onset of effect for the caregivers and improvement in ADHD symptoms (clinician assessment) for the children/adolescents, and the rank order continued to differ slightly for all of the other attributes, although never by more than two categories. Thus, preference for attributes of products and overall product profiles was similar between caregivers and their children/adolescents.

**Figure 3.** Mean Percent Importance Ratings for Attributes among Children/Adolescents

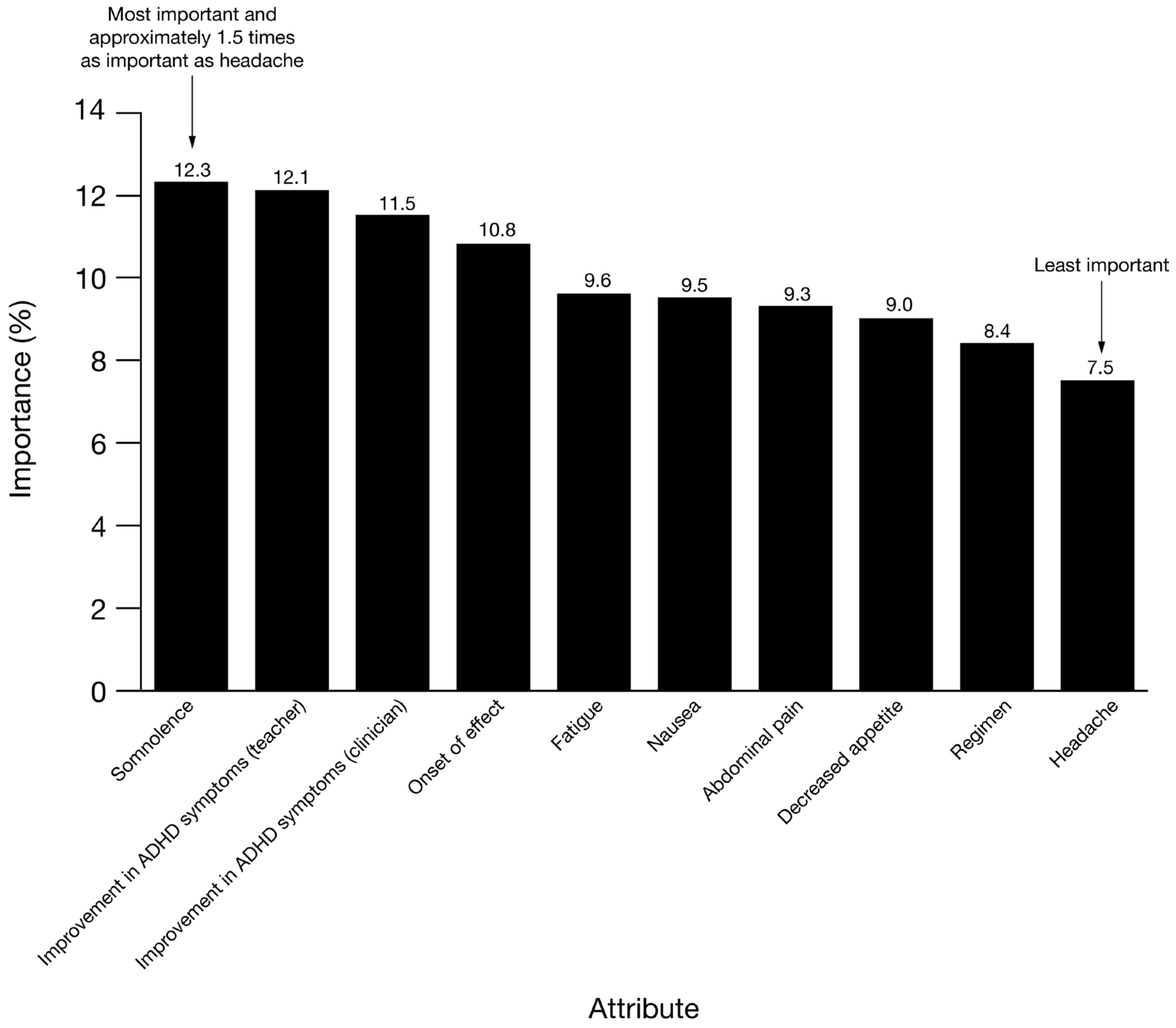

ADHD: attention-deficit/hyperactivity disorder

**Table 3.** Estimated Percentage of Caregivers and Children/Adolescents Preferring GXR over ATX (BaseCase and Sensitivity Analyses)

| | Caregivers | | Children/ Adolescents | |
|---|---|---|---|---|
| **Sample** | **GXR** | **ATX** | **GXR** | **ATX** |
| Base-case | 95.3% | 4.7% | 93.8% | 6.3% |
| Equal efficacy (doctor and teacher assessment) | 89.2% | 10.8% | 61.8% | 38.2% |
| Equal black box warning | 89.0% | 11.0% | – | – |
| Equal risk of abdominal pain | 85.4% | 14.6% | 70.2% | 29.8% |
| Equal risk of decreased appetite | 85.4% | 14.6% | 73.6% | 26.4% |
| Equal efficacy, best case for ATX and worst case for GXR | 26.3% | 73.7% | 39.7% | 60.3% |
| Side-effect profile (all attributes constant except side effects) | 93.6% | 6.4% | 61.8% | 38.2% |
| GXR has black box warning; ATX does not | 48.8% | 51.2% | – | – |
| Both GXR and ATX work "moderately well" (doctor) | 93.1% | 6.9% | 83.7% | 16.3% |
| GXR works "moderately well"; ATX works "very well" (doctor) | 77.3% | 22.8% | 66.3% | 33.7% |
| Both GXR and ATX work "moderately well" (teacher) | 93.9% | 6.1% | 83.2% | 16.8% |
| GXR works "moderately well"; ATX works "very well" (teacher) | 74.6% | 25.4% | 71.4% | 28.6% |
| GXR takes "4–8 weeks" to start working; ATX takes "2–4 weeks" | 91.0% | 9.1% | 84.4% | 15.6% |
| GXR takes "4–8 weeks" to start working; ATX takes "up to 2 weeks" | 84.0% | 16.0% | 63.5% | 36.5% |
| GXR has 45% chance of somnolence; ATX has 11% | 93.3% | 6.7% | 91.2% | 8.8% |

ATX: atomoxetine; GXR: guanfacine extended release

**Table 4.** Comparing Rank Order of Importance among Attributes Common to both Caregiver and Child/Adolescent Surveys

| Attribute | Caregiver Rank[a] | Child/Adolescent Rank[a] |
|---|---|---|
| Chance of feeling sleepy during the day | 1 | 1 |
| Improvement in ADHD symptoms (teacher assessment) | 2 | 2 |
| Onset of effect | 3 | 4 |
| Improvement in ADHD symptoms (clinician assessment) | 4 | 3 |
| Chance of abdominal pain | 5 | 7 |
| Chance of feeling tired or worn out | 6 | 5 |
| Chance of nausea | 7 | 6 |
| Chance of headache | 8 | 10 |
| Chance of decreased appetite | 9 | 8 |
| Regimen | 10 | 9 |

[a]1 = most important; 10 = least important; ADHD: attention-deficit/hyperactivity disorder

## DISCUSSION

This study identified the most important attributes in driving overall medication preferences for non-stimulant ADHD medications among children/adolescents with ADHD and their caregivers. This study also evaluated preference for GXR versus ATX based on these treatments' overall product profiles; to our knowledge, this study was the first to specifically examine preferences for non-stimulant medications and to elicit preferences directly from children. The results suggest that both children/adolescents with ADHD and their caregivers can express clear preferences for various medication attributes, including those related to hypothetical teacher- and clinician-perceived efficacy, caregiver-perceived improvement in oppositional behavior, regimen, and side effects associated with the two common non-stimulants. Among the general medication attributes evaluated in this study, those that were most influential in driving caregiver preferences for any treatment were risk of somnolence and effect on oppositionality, followed by absence/presence of a black box warning and other attributes related to efficacy, such as the improvement in ADHD symptoms hypothetically reported by both teacher and clinician and onset of effect. In general, besides the risk of somnolence, non-serious side effects such as fatigue, nausea, and headache were less important drivers of preference. While neither GXR nor ATX is indicated to treat oppositionality, the emergence of this attribute as an important general driver of preference suggests that effective treatment of comorbid conditions common to ADHD is at least equally as important to caregivers as the treatment of core symptoms.

This clear expression of preferences for various attributes of ADHD treatments, and particularly those attributes associated with efficacy, is consistent with previous preference research among caregivers of ADHD patients. For example, a recent study that utilized a mixed-methods approach (both a qualitative and a quantitative assessment) in a sample of 121 caregivers also showed that, among a range of attributes comprising both efficacy and side effects, the attributes valued as most important were improvements in the child's symptoms, social situation, and emotional state.[35]

When comparing GXR versus ATX, given base-case values of safety and efficacy attributes, a higher proportion of caregivers were estimated to prefer GXR among the two non-stimulant options. This preference was largely driven by a more favorable safety and efficacy based on clinician and teacher rating. The difference in efficacy in favor of GXR over ATX has been supported by literature.[29,36] Generally, GXR remained the favored product within both samples even when individual modifications were made to attribute level values in sensitivity analyses. This research suggests that children/adolescents and caregivers can express clear preferences for treatment attributes and, as such, should be active participants in their healthcare plans, which is supported by the shared decision-making model. Furthermore, studies have demonstrated that involving patients and caregivers in treatment decisions and goal setting can markedly improve adherence and overall health outcomes.[7,37]

This study had certain strengths and limitations. A key strength was that our samples represented a wide range of demographic characteristics, ADHD severity levels, treatment experience, and comorbidity burden; generally, we found no significant differences in preferences, based on patient demographic and clinical features. Additionally, the sex distribution of children/adolescents with ADHD in our study (70% male, 30% female) matches closely with the estimated nationwide diagnosed male-to-female ratio of 4:1.[38] These observations greatly contribute to the generalizability of our findings to the studied and diagnosed population of patients with ADHD. Moreover, our patient/caregiver-centered focus on the relative importance of attributes of non-stimulant medications provides an original within-class assessment of drivers of preference for medications that can be used as alternatives to stimulants.

One limitation is that the incidence of comorbidity was low for children/adolescents diagnosed with ADHD; half of children/adolescents were not diagnosed with any of the conditions that often accompany ADHD. This characteristic of the sample may have influenced the ADHD severity ratings and may limit the generalizability of our results. Another limitation is that we relied on caregiver self-report of child/adolescent ADHD diagnosis, which could potentially bias our results. Other limitations to the generalizability of our findings were that the caregiver sample in our study was highly educated compared to the general population (84% of caregivers in our sample had completed at least some college), and participation required internet access. It is possible that a less educated sample, or a sample without access to the internet, might express different preferences. However, in a subgroup analysis comparing caregivers with a bachelor's degree to caregivers without a bachelor's degree, no significant differences in preference were observed. It also should be noted that, although a wide range of medications were reported as being used currently or in the past, a fairly low proportion of children/adolescents had experience with the two non-stimulants being evaluated in this study. Thus, the preferences elicited with this sample were generally not based on experience, but instead, reflect real-world treatment decisions that are often made based on available information about the products rather than actual experience. It is possible that personal experience with these two products could influence caregiver and child/adolescent preferences. Another limitation of this study is that preferences expressed in this study do not account for all of the possible factors that could impact real-world decisions regarding treatment choice, such as cost, cultural beliefs, and physician influence. Moreover, the attributes included in the survey were limited to those for which credible comparative data were available.

## CONCLUSIONS

Children/adolescents and their caregivers have specific preferences with respect to non-stimulant ADHD treatment; these preferences should be weighed and considered by healthcare practitioners during treatment decisions. Specifically, side effects with a high risk of occurring or that are serious in nature, along with attributes related to efficacy, can be key drivers of preference for an ADHD non-stimulant medication. Caregivers also consider improvement in oppositionality, although not a treatment benefit appearing in current product labels, to be a key driver of preference. Similarly, children/adolescents were willing to accept a high risk of somnolence for better efficacy and quicker time to onset. Thus, healthcare providers should consider involving patients and caregivers in the medical decision-making process by both sharing relevant information about product characteristics and general patient and caregiver preferences, and incorporating stated preferences into treatment plans for children with ADHD. This patient-centered approach may result in a greater concordance regarding treatment goals and treatment choice that could lead to better, adherence, and clinical and quality-of-life outcomes.

## ACKNOWLEDGMENTS

This study was funded by Shire Development, LLC. Although the funding body was involved in the design, collection, analysis, interpretation, and fact checking of information, the content of this manuscript, the ultimate interpretation, and the decision to submit it for publication in Journal of Health Economic and Outcomes Research was made by the authors independently. Jessica Grinspan is thanked for her work on the study and the manuscript whilst employed by ICON PRO. Editorial assistance was provided by Caudex, funded by Shire International GmbH.

### Conflict of Interest Declaration

EF is an employee of ICON PRO, which received funding from Shire Development, LLC, for performing the analyses and drafting the manuscript. KG, VS, PH, and MHE were, at the time of the study, employees of Shire. FL is a consultant for and has received grant support from Shire Pharmaceuticals. DC is a consultant for Shire Pharmaceuticals and Rhodes Pharmaceuticals and has received grant support from Shire Pharmaceuticals.

A preliminary version of this study was presented as a poster at the American Academy of Child and Adolescent Psychiatry meeting in 2013.
